# Developmental regulation of fetal mitochondrial respiratory function towards term: the role of glucocorticoid and thyroid hormones

**DOI:** 10.1530/JOE-25-0135

**Published:** 2025-10-18

**Authors:** A L Fowden, K L Davies, E J Camm, A J Forhead, A J Murray

**Affiliations:** ^1^ Department of Physiology, Development and Neuroscience, University of Cambridge, Cambridge, UK; ^2^ Hudson Institute of Medical Research, Clayton, Victoria, Australia; ^3^ Department of Biological and Medical Sciences, Oxford Brookes University, Oxford, UK

**Keywords:** mitochondria, glucocorticoids, thyroid hormones, oxidative phosphorylation

## Abstract

Mitochondria are unique intracellular organelles that have their own DNA and are inherited intact in the oocyte. They have multiple functions, the most important of which is producing energy in the form of ATP by oxidative phosphorylation (OXPHOS) using a range of metabolic substrates. As energy requirements increase with intrauterine growth and the onset of new postnatal functions at birth, mitochondria develop structurally and functionally *in utero* to meet these energy demands. In part, the developmental and prepartum maturational changes in mitochondrial OXPHOS capacity depend on the endocrine environment and the natural rise in the fetal concentrations of hormones, such as cortisol and tri-iodothyronine (T_3_), towards term. This review discusses the development of mitochondrial respiratory function during late gestation with an emphasis on tissue OXPHOS capacity. It considers the role of cortisol and thyroid hormones, in particular, in the intrauterine development and prepartum maturation of mitochondrial OXPHOS capacity in preparation for extrauterine life. Finally, it briefly examines the potential longer-term consequences of abnormal hormonal exposure before birth on mitochondrial OXPHOS function later in postnatal life. Endocrine regulation of mitochondrial OXPHOS in the fetus is shown to be multifactorial, dynamic and tissue specific with a central role in determining functional development. It optimises energetics for survival both *in utero* and at birth and has implications for adult metabolic fitness and the inheritance of mitochondrial phenotype.

## Introduction

Mitochondria are widely held to have arisen from the endosymbiotic incorporation of bacterial genomes into early eukaryotic cells (1). They retain their own DNA (mtDNA) which, together with the nuclear DNA, regulates mitochondrial structure and function. Unlike nuclear DNA, mtDNA is circular in structure and has no histones. It contains 37 genes, 13 of which code proteins in humans (2). Intact mitochondria and mtDNA are inherited through the maternal line in the oocyte and, hence, they act as markers of ancestry and evolutionary influences across the generations (1). Classically, mitochondria have been portrayed as discrete organelles; however, they are now recognised to be dynamic entities that can form a reticulum and show a wide range of morphologies across different tissues that can undergo structural and functional changes in response to the stimuli (3, 4). In adulthood, mitochondria respond to energy demand and environmental cues through biogenesis, fusion/fission and alterations in their biochemical and protein composition (5, 6). Damaged mitochondria undergo selective autophagy (termed mitophagy) and clearance as a means of quality control with growing evidence for the involvement of mitophagy in mitochondrial turnover and cellular metabolic remodelling during processes such as early embryonic development and cell differentiation (7, 8, 9). Alterations in mitochondrial abundance, structure, substrate metabolism and respiratory function, therefore, play a critical role in the adaptive response of healthy tissues to changing environmental conditions, whilst an inability or failure of the mitochondria to respond appropriately or sufficiently to environmental cues can challenge tissue energy homoeostasis and lead to ill health (4). In addition, inherited mutations in mtDNA can give rise to pathologies, collectively termed mitochondrial disease, and these can be severe in nature (5, 9).

Hormones are known to play important roles in metabolic adaptation to environmental cues in adulthood. In particular, glucocorticoid and thyroid hormones have been shown to influence adult mitochondrial phenotype and energetics, often in a tissue specific manner (5, 10). Mitochondrial pathways are intimately connected with cell differentiation and growth, and notably both glucocorticoids and thyroid hormones support anaplerotic flux in adult cells and tissues, replenishing tricarboxylic acid (TCA) cycle intermediates in support of biosynthetic function (11, 12). These hormones also have a central role in the control of prenatal metabolism by regulating the supply and utilisation of metabolic substrates by the feto–placental tissues (13, 14). Glucocorticoids, in particular, act as environmental and maturational signals *in utero*, altering tissue growth and differentiation. They act both directly and indirectly by controlling the fetal availability of other metabolic and growth regulatory hormones, such as leptin, the insulin-like growth factors, adrenaline and T_3_ (15). Recent studies have indicated that the glucocorticoids and thyroid hormones are also involved in the structural and functional development of feto–placental mitochondria. This review, therefore, examines the developmental regulation of mitochondrial respiratory function, with particular emphasis on the role of the glucocorticoids and thyroid hormones in the maturation of OXPHOS capacity in feto–placental tissues towards term.

## Mitochondrial structure and function

**Figure 1:**
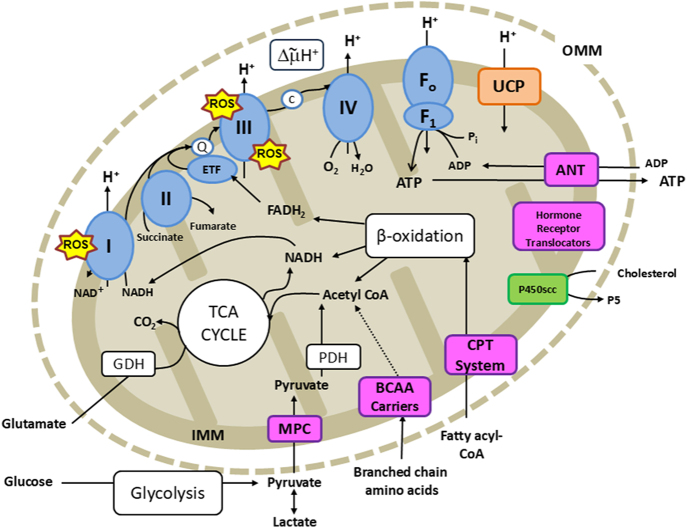
Schematic diagram showing the mitochondrial electon transfer system (ETS) and metabolic pathways contributing to the production of ATP and steroids. OMM = outer mitochondrial membrane. IMM = inner mitochondrial membrane. The blue ovals and circles represent the ETS comprising complexes I, II, II, IV and F1/Fo ATP synthase (also known as CV), electron transfer flavoprotein (ETF), Coenzyme Q (Q, also known as ubiquinone) and cytochrome c (c). The pink boxes represent the transmembrane transporter and carrier molecules as follows: ANT = adenine nucleotide transporter, CPT system = carnitine palmitoyl transferase, BCAA carriers = branched chain amino acids carriers, MPC = mitochondrial pyruvate carrier, and the hormone receptor transporters. The yellow stars indicate the sites of ROS production within the ETS, and the uncoupling proteins (UCP) are represented with an orange box. The white boxes show metabolic processes, including the tricarboxylic acid (TCA) cycle, beta-oxidation, glycolysis, plus the enzymes pyruvate dehydrogenase (PDH) and glutamate dehydrogenase (GDH), and the H+ electrochemical gradient across the IMM (ΔμH+). The green box shows the enzyme P450 side chain cleavage (P450scc), which produces pregnenolone (P5) from cholesterol in steroidogenic tissues.

Mitochondria are double-membraned structures, with an outer membrane and an inner membrane separated by the intermembrane space (Fig. 1). A proton gradient across the inner mitochondrial membrane (IMM) gives rise to a potential difference between the intermembrane space of the mitochondrial matrix that is important for several mitochondrial functions (16). To maximise the surface area of the IMM, it is folded into finger-like projections, termed cristae (17). The cristae differ in density, shape and size between different tissues, with environmental conditions and during development (18). At the ultrastructural level, the cristae can appear tubular or vesicular depending on the specific tissue and its functional status. The IMM houses the electron transfer system (ETS) and other biomolecules regulating the production of energy through oxidative phosphorylation of ADP to ATP (Fig. 1). In certain endocrine tissues, such as the gonads, adrenal cortex and placenta, the IMM also houses the key enzymes required for steroidogenesis (19). In addition, the IMM contains transporters and ion channels specific to particular functions. In comparison, the outer mitochondrial membrane is relatively porous, although its structural integrity is essential for normal mitochondrial function. The mitochondrial matrix contains mtDNA, ribosomes, and most enzymes of the TCA cycle, beta-oxidation and other pathways of substrate metabolism that provide reducing equivalents to the ETS (2).

A major function of mitochondria in most tissues is ATP production via OXPHOS, which is reliant on the ETS. This system is composed of protein complexes (C), each comprising multiple proteins encoded primarily by the nuclear genome, but with mtDNA-encoded proteins incorporated into CI, CIII and CIV. Dehydrogenase enzymes of the ETS catalyse redox reactions with electrons transferred from NADH at CI, and succinate at CII to ubiquinone/ubiquinol, with electron transfer converging at the Q-cycle of CIII (Fig. 1). Other mediators of electron transfer to ubiquinol include the electron-transferring flavoprotein (ETF) dehydrogenase, which connects fatty acid oxidation with the ETS. From CIII, electrons are carried by cytochrome c to react with oxygen (O_2_) at CIV, producing water. At CI, CIII and CIV, redox reactions generate protons, which are pumped across the IMM generating the proton gradient (Fig. 1). The protonmotive force across the IMM is used to drive the formation of ATP through ADP phosphorylation at the F_1_F_o_-ATP synthase, also called CV (Fig. 1). The efficiency of OXPHOS is dependent on proton leak across the IMM, which may be mediated by proteins, such as the uncoupling proteins (UCPs) and/or the adenine nucleotide translocase (ANT). ANT also shuttles ADP and ATP into and out of the mitochondria, respectively (Fig. 1). The ETS complexes undergo higher-level organisation into the so-called supercomplexes, such as the respirasome, which comprises CI_1_ CIII_2_ and CIV_1_ (3). This is a dynamic process and has been proposed to have functional consequences by enhancing electron transfer efficiency (6). Electron donors to the ETS (including NADH, succinate, and acyl-CoA) are derived from the oxidation of substrates, such as pyruvate, amino acids, fatty acids and ketone bodies. Consequently, substrate availability is an important determinant of ATP production and varies with diet, fuel reserves and, in fetal somatic tissues, with the stage of development and with the transport and metabolism of nutrients by the placenta. In addition, availability of O_2_, the final electron acceptor, is a determinant of OXPHOS capacity, with the consequence that chronically low O_2_ levels can impact tissue mitochondrial content, distribution, ETS complex abundance, OXPHOS efficiency and substrate preference (16).

Mitochondria have multiple other functions, in addition to ATP production, which can be tissue specific (9). At complexes I and III, mitochondria generate reactive oxygen species (ROS), which act as signalling molecules influencing mitophagy and cellular differentiation, senescence and death under both normal and adverse environmental conditions (4). When the proton gradient is high or O_2_ availability is limited, electrons are diverted from the ETS to produce superoxide anions (O_2_^−^) and hydrogen peroxide (H_2_O_2_) (Fig. 1). Mitochondria also play a number of tissue-specific roles to support biosynthesis. In steroid producing cells, mitochondria control the initial step in steroidogenesis, the production of pregnenolone (P5) from free cholesterol derived from the cytoplasm using specific transporters in the mitochondrial membranes (Fig. 1). Depending on the specific endocrine tissue, the IMM can also contain additional enzymes further down the steroidogenic pathways required, for instance, to produce cortisol (20). In the liver, mitochondria support ketogenesis during undernutrition, with acetoacetate and β-hydroxybutyrate generated from fatty acid-derived acyl-CoA. Mitochondrial pathways, including the TCA cycle, are central to many biosynthetic pathways, including steroidogenesis, ketogenesis and gluconeogenesis. As such, the regulation of both anaplerotic and cataplerotic reactions is critical to balance the substrate availability and maintain flux to support biosynthetic function (21). When activated by sympathetic stimulation, brown and beige adipose tissue generate heat via non-shivering thermogenesis, owing to their high mitochondrial content and the presence of UCP1, which uncouples fatty acid oxidation and electron transfer from ATP synthesis (Fig. 1). Furthermore, mitochondria can influence cellular calcium (Ca^2^^+^) homoeostasis by changes in the abundance of their membrane Ca^2+^ channels and transporters, thereby altering the Ca^2+^ distribution between the mitochondria matrix and other subcellular organelles, such as the endoplasmic and sarcoplasmic reticula (22). These mitochondrial Ca^2+^ movements also affect the potential gradient across the IMM, with consequences for other mitochondrial functions, including OXPHOS, whilst matrix Ca^2+^ activates TCA cycle dehydrogenase enzymes.

## Prenatal development of mitochondrial OXPHOS function

As fetal mass and placental nutrient transfer rise with development, energy is required in increasing amounts. During early mammalian development, O_2_ availability *in utero* is low and the embryo is largely reliant on glycolysis for ATP production (23). When O_2_ and nutrient availability increase with the formation of the placenta, mitochondrial OXPHOS becomes a more prominent source of ATP for the feto–placental tissues. In most fetal tissues, mitochondrial OXPHOS provides the majority of the ATP requirement by term (24). The metabolic switch from glycolysis to aerobic mitochondrial OXPHOS allows more ATP to be generated per molecule of glucose, sparing glucose carbon for the accretion of new fetal tissue. At birth, energy requirements increase again with the onset of new postnatal functions essential for survival *ex utero* (25). These include breathing, glucoregulation, thermogenesis, digestion, renal salt and water balance, and, in precocial species, locomotion. The increases in postnatal energy requirements occur concomitantly with the greater atmospheric O_2_ content and a switch in the metabolic substrates from using mainly carbohydrates *in utero* to a more fat-based diet of milk after birth (15, 17, 26). To support these developmental changes in energy demand, mitochondria adapt their morphology and function in line with the changing availability of O_2_ and oxidative substrates by biogenesis, fusion, fission and mitophagy.

In most tissues studied in the fetus, mitochondrial biogenesis occurs with increasing gestational age, driven, in part, by the upregulated expression of the *PGC1a* gene encoding the protein, PGC-1α, the transcriptional coactivator peroxisome proliferator-activated receptor 1α, (27). This leads to an increment in the cellular mitochondrial content, particularly during the perinatal period in tissues, such as the liver and skeletal muscles, in which energy demands rise rapidly at birth (Table 1). Increases in mitochondrial content are also observed in the heart of fetal sheep and in the heart, liver and kidney of fetal rat pups close to term (34, 35, 36). The absolute mitochondrial content and the magnitude and timing of the perinatal increment vary between different tissues in fetal sheep and rats (Table 1, (35)). In a range of human tissues, there are also marked increases in the mitochondrial content and respiratory activity between the second trimester and early postnatal life (37). In addition, mitochondria elongate during intrauterine development by mitochondrial fusion regulated, in part, by the mitofusin proteins, encoded by the *MFN* genes (17, 18). Furthermore, the cristae develop a more laminar structure and expand in number and complexity with increasing gestational age (18). Towards term in several species, mitochondria, therefore, appear more vesicular or tubular depending on their orientation, particularly in tissues that become more active prenatally, such as the adrenal cortex, or switch from growth to differentiation in preparation for their new metabolic functions after birth, such as the liver, cardiac and skeletal muscles (17, 38, 39, 40, 41).

**Table 1:** Tissue mitochondrial content, measured as citrate synthase activity, and plasma cortisol and thyroid hormone concentrations in fetal sheep during the perinatal period. Term: approximately 145 days, 130 days = 90% gestation, 143 days = 98% gestation.

	Developmental age		
	130 days	143 days	Newborn
**Citrate synthase (nmol/min/mg protein)**			
Muscles			
* Semitendinosus* (*28*)^*†*^	137 ± 18^a^	274 ± 25^b^	460 ± 31^c^
*Biceps femoris* (*29*, *30*)	163 ± 11^a^	210 ± 26^b^	350 ± 10^c^
*Superficial digital flexor* (*30*)	120 ± 10^a^	180 ± 30^a,b^	260 ± 30^b^
* Soleus^†^*	69 ± 15^a^	161 ± 28^b^	268 ± 16^c^
Liver^†^	36 ± 2^a^	56 ± 5^b^	47 ± 5^b^
Perirenal adipose tissue (31)	620 ± 60	920 ± 90*	NA
Cerebral cortex (32, 33)	160 ± 10	250 ± 10*	NA
Cerebellum (32, 33)	200 ± 15	230 ± 20	NA
**Hormones (ng/mL)**			
Cortisol	13.8 ± 2.1^a^	58.7 ± 9.8^b^	121.7 ± 26.0^c^
Tri-iodothyronine	0.38 ± 0.03^a^	0.90 ± 0.05^b^	4.01 ± 0.34^c^
Thyroxine	104.7 ± 11.9	98.0 ± 19.0	83.1 ± 7.9

Citrate synthase values are the means (±SEM) for individual animals in cohorts from one or more publications indicated by the reference number as a superscript, or are unpublished observations by KL Davies and AL Fowden, and indicated by the † symbol by the relevant tissue. NA = not available. At each age, hormone concentrations are the mean (±SEM) values for all animals that provided tissue for the measurement of citrate synthase activity. The values with different letters are significantly different from each other, *P* < 0.05, one-way ANOVA.* Significantly different from the value at 130 days, *P* < 0.05, *t*-test. The unpublished citrate synthase values used archival tissue from animals reported in references (28, 29, 30, 31, 32, 33). All citrate synthase and hormone concentrations were measured as described in reference (29).

**Figure 2:**
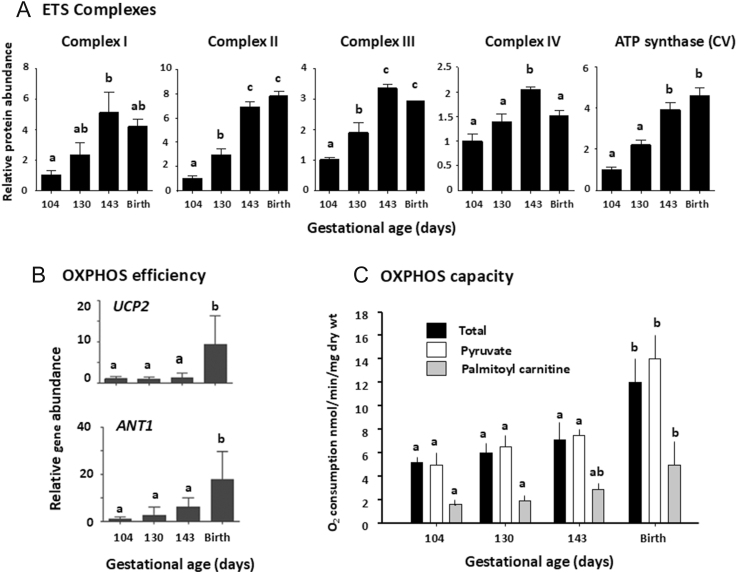
The mean (±SEM) values of (A) protein abundance of the ETS complexes I to IV and ATP synthase (CV). (B) Gene expression of uncoupling protein 2 (*UCP2*) and adenine transporter 1 (*ANT1*) that affect the efficiency of oxidative phosphorylation (OXPHOS). (C) Rates of the total OXPHOS (black columns) and OXPHOS supported specifically by pyruvate (open columns) or palmitoyl-carnitine (grey columns) in the *biceps femoris* muscle of fetal sheep with respect to gestational age. Term ≥145 days. For each protein, gene and OXPHOS rate, values with different letters are significantly different from each other with respect to the gestational age (one way ANOVA, *P* < 0.05). Data from references (29, 30).

These gestational alterations in mitochondrial morphology are accompanied by functional changes in the ETS and OXPHOS capacity in several fetal tissues. In ovine fetal skeletal muscles, the abundance of ETS complexes and ATP-synthase increases perinatally, peaking either just before or at birth, with increments in the mitochondrial ANT and UCP gene expression only after birth (Fig. 2A and B; (29, 30)). Similar prenatal increases are seen in the abundance of complexes I and IV in the cerebral cortex of fetal sheep and in complex IV in fetal rat kidneys and liver (32, 33, 34, 35). In general, the increased ETS complex abundance parallels the rise in mitochondrial content and cristae lamination (Table 1; (17)). In skeletal muscles, this may relate, in part, to the developmental changes in muscle fibre composition and in the relative proportion of the intermyofibrillar and subsarcolemmal mitochondria within the total muscle mitochondrial population (29, 42). In the ovine fetal brain, rising mitochondrial density and ETS complex abundance towards term are associated with increased OXPHOS capacity in a region and substrate specific manner (32). In contrast, in skeletal muscle, which accounts for a greater proportion of the fetal mass and total oxygen consumption than the brain, there appears to be little change in the mass-specific OXPHOS capacity either in total or using specific metabolic substrates until after birth, despite the prenatal increases in the mitochondrial content and ETS complex abundances (Table 1, Fig. 2, (29)). Consequently, the OXPHOS capacity per mitochondrion declines towards term in ovine skeletal muscles (29), consistent with the maintained total rate of O_2_ consumption per kg fetus up until birth (43, 44). However, the total and substrate specific OXPHOS capacity of the skeletal muscle doubles immediately at birth (Fig. 2C), in line with the rapid rise in the total O_2_ consumption by the newborn lamb (43). Similarly, at birth, there is a 2–5-fold increase in the mitochondrial OXPHOS capacity of the kidney and liver of newborn piglets and rat pups, respectively, which continue to rise over the immediate neonatal period (35, 42). Fetal tissues, such as skeletal muscle, therefore, appear to increase their capacity for mitochondrial ATP production in anticipation of birth by increasing the mitochondrial content but only activate additional OXPHOS after delivery has occurred. This strategy may prevent hypoxia developing *in utero* while meeting the high demands for energy and heat immediately after birth. In addition, by upregulating muscle UCP abundance only at birth, ATP production remains efficient *in utero* while limiting excessive ROS production neonatally and, hence, potential tissue damage, when OXPHOS activity and O_2_ availability increase rapidly after birth.

The gestational changes in the placental mitochondrial OXPHOS appear to differ from those seen in the somatic tissues of the fetus *per se*. In the mouse placenta, the content and OXPHOS capacity of the mitochondria initially increase from mid-gestation to late gestation but then decrease close to term, in a manner that depends on the placental zone and on the sex and size of the fetus (45, 46, 47). Overall, the prepartum reduction in mitochondrial content is more pronounced in the endocrine than in the nutrient transport zone of the placenta, whereas a late gestation decrease in the complex IV is more evident in the transport zone of the mouse placenta, which may account for the lower respiratory capacity in the absence of any change in the mitochondrial content (45). Despite this fall in the overall OXPHOS capacity, there was relative preservation of fatty acid supported respiratory capacity in the late gestation compared with pyruvate-supported respiration, in part, due to lower leak respiration with fatty acid than carbohydrate substrates (45). This might spare glucose for the transport to the fetus when it is growing rapidly in absolute terms.

Near term, mitochondrial OXPHOS efficiency in the transport zone also appears to be greater in the lightest than in the heaviest female pups in a litter as the CI + CII-supported OXPHOS was greater in the lightest females, despite lower CI abundance in the lightest females (47). In male littermates, there was no difference in the placental OXPHOS capacity with fetal body weight, although complexes II and IV were lower in abundance in the lighter than heavier males. The two sexes, therefore, appear to adopt different metabolic strategies to maintaining placental OXPHOS capacity as the fetus grows towards term. The sex and body weight differences in OXPHOS capacity may relate, in part, to the innate differences in steroid exposure between the sexes and to the altered placental steroidogenic gene expression observed with reduced body weight in female but not male littermates (47). They could also reflect the sex and growth linked differences in the abundance of androgen and oestrogen receptors known to be present in mitochondria (4). Collectively, these observations suggest that placental mitochondria age metabolically towards term but can also adapt functionally to support the fetal growth demands during late gestation, in a manner that appears to be sex-linked (47, 48).

## Endocrine regulation of prenatal maturation of mitochondrial OXPHOS

In adulthood, tissue metabolism, including mitochondrial respiration, is responsive to a range of hormones, including the glucocorticoids, thyroid hormones and sex hormones. These hormones act via non-genomic and genomic mechanisms to impact mitochondrial biogenesis, dynamics and function (49). They influence the activity of key rate-limiting metabolic enzymes and regulate the transcription of mitochondrial genes in both the nuclear and mitochondrial DNA (4, 9). Mitochondria have sequences in their DNA that resemble the hormone response elements of the nuclear DNA (50, 51) and can respond to the hormones either via receptors located in their IMM and matrix or by importing the receptor bound hormone from the cytoplasm (52, 53, 54, 55). While thyroid hormone receptors (THRs) appear to be localised on the IMM, mitochondrial glucocorticoid receptors (GRs) are probably derived from the cytoplasm through channels formed by translocator complexes located in the mitochondrial membranes, using energy derived from the ATP and the proton gradient across the IMM (55). The GRs can then bind to the glucocorticoid response elements in the mtDNA and initiate transcription of the mitochondrial ribosomal RNA followed by the translation of specific subunits of the ETS protein complexes (49, 50, 56, 57). In adult tissues, glucocorticoid and thyroid hormones are, therefore, key environmental regulators of OXPHOS through transcriptional actions on the OXPHOS genes and via non-genomic alterations in the OXPHOS substrate availability.

Prenatal maturation of the key fetal tissues essential for neonatal survival depends on the natural rise in fetal glucocorticoid concentrations caused by the increased adrenal secretion of glucocorticoids towards term (25). In turn, this increment in glucocorticoid concentrations leads to increases in the circulating T_3_ concentration and in tissue T_3_ availability, in part, by the glucocorticoid-dependent activation of specific thyroid hormone deiodinases in a tissue specific manner ((13); Table 1). The prepartum maturational events critical for survival at birth are, therefore, dependent on both glucocorticoid and thyroid hormones. In precocial species, such as ruminants and pigs, the major glucocorticoid increment is prenatal, peaking at birth, whereas in atrial species, such as rodents, the prepartum increase in glucocorticoid concentrations is less pronounced with the main increment occurring in the days following birth (25). Consequently, species differences exist in the profile of glucocorticoid-dependent maturational events and in the metabolic responses to glucocorticoid overexposure *in utero*.

### Glucocorticoids

**Figure 3:**
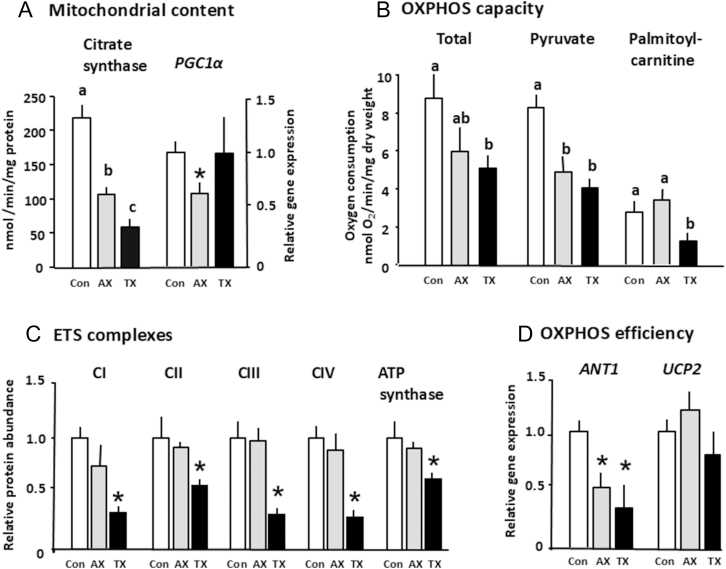
The mean (±SEM) values of (A) mitochondrial density measured as citrate synthase activity and gene expression of *PGC1α*, (B) oxidative phosphorylation (OXPHOS) capacity measured as rates of total, pyruvate and palmitoyl-carnitine supported OXPHOS, (C) protein abundance of the electron transfer (ETS) complexes (C) I to IV and ATP synthase (CV) and (D) gene expression of uncoupling protein 2 (*UCP2*) and adenine transporter 1 (*ANT1*) that affect OXPHOS efficiency in the *biceps femoris* of sheep fetuses at 143 days of gestation that were sham operated as controls (Con, open columns), adrenalectomised (AX, grey columns) or thyroidectomised (TX, black columns) earlier in gestation. Term ≥145 days. Values with different letters are significantly different from each other with respect to treatment (one way ANOVA, *P* < 0.05). *Significantly different from the control value *P* < 0.01 (*t*-test). Data from references (29,30).

**Figure 4:**
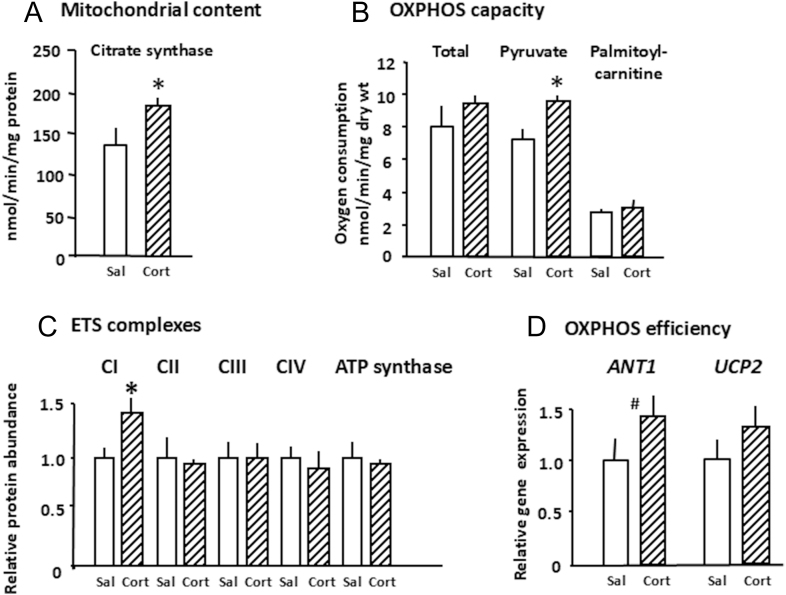
The mean (±SEM) values of (A) mitochondrial density measured as citrate synthase activity, (B) oxidative phosphorylation (OXPHOS) capacity measured as rates of total, pyruvate and palmitoyl-carnitine supported OXPHOS, (C) protein abundance of the electron transfer (ETS) complexes I to IV and ATP synthase (CV) and (D) gene expression of uncoupling protein 2 (*UCP2*) and adenine transporter 1 (*ANT1*) that affect OXPHOS efficiency in the *biceps femoris* of sheep fetuses at 130 days of gestation after infusing saline as a control (Sal, open columns) or cortisol (Cort, Stripped columns) for the previous 5 days. Term ≥145 days *Significantly different from the saline infused control value *P* < 0.01, # Trend, *P* < 0.10, (*t*-test). Data from references (29, 30).

In fetal sheep, the prepartum glucocorticoid increment closely parallels the increases in mitochondrial content and ETS complexes seen over the last 5–10% of gestation in tissues, such as the skeletal muscle, adipose tissue, liver, heart and brain (Table 1, (36)). When the cortisol increment is abolished by surgically removing the adrenal glands in fetal sheep, mitochondrial content, *PGC1α* and *ANT1* expression and the total and Py-supported OXPHOS capacity are lower in specific skeletal muscles at term than in the sham-operated controls (Fig. 3; (30)). This occurs with only minor, if any, changes in abundance of the ETS complexes (Fig. 3C), possibly suggesting an impact on supercomplex formation. Conversely, short term cortisol infusion into fetal sheep just before the onset of the normal prepartum cortisol increment at 90% gestation leads to premature increases in the mitochondrial content, *PGC1α* expression and OXPHOS capacity in the brain and specific skeletal muscles and in UCP1 in the adipose tissue (Fig. 4; Table 2; (30, 33, 59)). Earlier in gestation (≈75% term), short term infusion of cortisol into the fetal sheep has also been shown to increase the cardiac mitochondrial complex I abundance (Table 2; (58)). The effects of manipulating cortisol levels in the fetal sheep in late gestation (90% term) are muscle specific with elevated CI abundance and pyruvate-supported OXPHOS in the *biceps femoris* (Fig. 4) but not in the *superior digital flexor*, while the total and fatty acid oxidation were higher than the control values solely in the *superior digital flexor* muscle (30). Similarly, in pregnant rats adrenalectomised and treated with metyrapone to prevent any prepartum rise in fetal corticosterone concentrations, CIV abundance was reduced in the fetal kidney and liver at term and restored to normal values when the pups also received short-term *in utero* treatment with the synthetic glucocorticoid, dexamethasone at 90% gestation (35).

**Table 2:** Effects of prenatal glucocorticoid exposure on mitochondrial OXPHOS function in feto–placental tissues.

	Species	Gestational age at treatment	Gestational age at study	Fetal tissue	Mitochondrial outcome	Reference
**Fetal treatment**						
*Synthetic*	Rat	16d	19d and 21d	Kidney	↑ Mitochondrial content	(35)
*Glucocorticoid*					↑ CIV gene expression	
*Dexamethasone*			19d	Liver	↑ CIV gene expression	
*Cortisol*	Sheep	109–116d	116d	Heart	↑CI gene expression and protein	(58)
		125–130d	130d	Skeletal muscle specific	↑ PGC1α	(30)
					↑ Mitochondrial content	
					↑ OXPHOS	
					↑ CI and ANT1 proteins	
					↑ Mitochondrial content	
				Cerebellum	↓ C1 OXPHOS	(32)
				Adipose tissue	↑ UCP protein	(59)
**Maternal treatment**						
*Synthetic glucocorticoids*	Mouse	14.5–20.5d	20.5d–birth	Adipose tissue	↓ PGC1α and mitochondrial content	(60)
*Dexamethasone*					↓ Mitochondrial cristae	
					↓ Cytochrome c	
					↓ UCP1 protein	
	Rat	14–16d	16d	Brain	↑ Maximal OXPHOS	(61)
		19–20d	21d	Heart	↑ ATP production	(62)
	Sheep	138d	140d	Adipose tissue	↑ UCP1 and 2 protein	(63)
					↑ VDAC protein	(64)
					↑ Cytochrome c	
*Corticosterone*	Mouse	12.5–14.5d	14.5d	Placenta	↓ Mitochondrial content	(65)
					↓ CIII protein	
		16.5–17.5d	17.5d	Heart	↑ OXPHOS, ↑PGC1α	(27)
					↑ Fatty acid OXPHOS capacity	(66)
*Cortisol*	Sheep	120–121d	122d	Heart	↓ *PGC1α* expression	(66)
		115–143d	143d	Heart	↓ Mitochondrial content	(67)
				Skeletal muscle	↓ Mitochondrial content	(68)
					Altered cristae formation	
					↓ CIV, ANT1 and OXPHOS genes	

UCP, uncoupling protein; VDAC, voltage-dependent anion channel; OXPHOS, oxidative phosphorylation, electron transfer system complexes CI to CV; MFN, mitofusin; PGC1α, peroxisome proliferator-activated receptor-γ coactivator; ANT, adenine nucleotide transporter; d, days gestational age.

In atricial species, such as rats and mice, maturational changes in mitochondrial function extend into the neonatal period in line with the continued rise in corticosterone concentrations after birth (35, 62, 69). Abolishing this postnatal increment by adrenalectomising the neonatal rat pups prevents the increase in ETS complex abundances and IMM surface density seen in renal mitochondria during the suckling period (69), in line with the glucocorticoid-induced changes in mitochondrial development seen prenatally in other, more precocial species. However, in mice, fetal corticosterone is required prenatally for the development of mitochondrial ultrastructure as fetuses with *Cyp11a1* gene deletion lack cristae in their adrenal mitochondria close to term (70).

Indeed, ontogenic changes to the structure and function of mitochondria in the adrenal zona fasciculata may be a contributory factor to the gestational rise in fetal glucocorticoid concentrations. The enzymes P450 side chain cleavage (P450scc) and 11-beta hydroxylase are co-localised to the mitochondrial IMM and control the first and last steps, respectively, in the biosynthesis of cortisol and corticosterone (19). Synthesis of pregnenolone (P5) by P450scc is known to be significantly higher in ovine neonatal than fetal adrenal cells (71), consistent with the increase in cellular mitochondrial content seen in other fetal tissues towards term (Table 1). Synthesis of P5 and activity of P450scc were also higher in the isolates of ovine neonatal than fetal mitochondria, which suggests that P450scc abundance may also increase per adrenal mitochondrion during the perinatal period (72). A positive feedback loop may, therefore, exist between the local cortisol production and adrenal mitochondrial content and/or steroidogenic capacity, which contributes to the prepartum increment in fetal cortisol concentrations and the consequent maturation of mitochondrial function in other fetal tissues towards term.

Maternal administration of glucocorticoids has also been shown to affect mitochondrial development in a range of fetal tissues across species (Table 2). In common with fetal manipulations, short term maternal administration of either natural or synthetic glucocorticoids close to term (≥90% gestation) increases the mitochondrial biogenesis and OXPHOS capacity in fetal tissues of several species (Table 2, (27, 62, 63)). However, when maternal cortisol treatment is prolonged in sheep (over the last 20% of gestation), mitochondrial density and OXPHOS gene expression tend to be reduced in fetal tissues (Table 2; (67, 68)). Similar reductions in mitochondrial OXPHOS capacity are seen in feto–placental tissues in response to more prolonged maternal treatment with dexamethasone or corticosterone in rodents (60, 65). Mitochondrial biogenesis and OXPHOS are also impaired in several fetal tissues of sheep and other species when fetal cortisol or corticosterone concentrations are raised for longer periods of gestation under adverse environmental conditions, including reduced uterine blood flow, hypoxia, maternal dietary manipulations, heat stress or placental insufficiency (73, 74, 75, 76, 77, 78, 79, 80, 81, 82, 83, 84, 85). In addition, these types of sub-optimal environmental conditions are known to have detrimental effects on the placental mitochondrial phenotype in several species, in addition to causing variations in O_2_ and nutrient availability directly (86, 87). Furthermore, dexamethasone has been shown to reduce mitochondrial activity in cultured human placental explants collected at term (88).

Collectively, the studies of prenatal glucocorticoid administration indicate that the dose, timing in gestation, type and duration of glucocorticoid exposure are all important determinants of the OXPHOS capacity of the feto–placental mitochondria (Table 2). GR abundance is also likely to be a contributory factor to the mitochondrial effects of glucocorticoid exposure as expression of the various GR isoforms varies between the feto–placental tissues and with gestational age and glucocorticoid exposure in a tissue specific manner (36, 69, 89). Furthermore, since glucocorticoid overexposure irrespective of its origin can lead to reductions in placental size and/or nutrient transfer (15), it remains unclear whether the actions of the glucocorticoids on fetal tissue mitochondria are direct or are mediated indirectly through altered availability of O_2_ and/or metabolic substrates. Indeed, glucocorticoid-induced reductions in the mitochondrial OXPHOS capacity of the placenta may impair ATP production for its active transport processes, consistent with the reduced transplacental transfer of amino acids seen in response to maternal corticosterone treatment of mice in late pregnancy (65, 90).

### Thyroid hormones

The tissue specificity of the mitochondrial responses to intrauterine glucocorticoid exposure suggests that there are additional factors involved in prenatal mitochondrial development. Thyroid hormones have a key role in regulating mitochondrial function in adulthood and are known to stimulate the growth and development of the fetus (10, 13). The placenta is more permeable to maternal thyroid than glucocorticoid hormones, particularly in human and rodent species that have a hemochorial placenta (13). In species with a multi-layered epitheliochorial placenta, such as sheep and pigs, the fetus receives less thyroid hormones from the mother and depends on its own thyroid gland for an adequate thyroxine (T_4_) supply (13, 42). In most species studied to date, the fetal T_3_ concentrations rise naturally towards term, independently of maternal concentrations, due to the glucocorticoid-stimulated upregulation of the tissue deiodinases that converts T_4_ to the more biologically active T_3_ (91). The prepartum plasma T_3_ surge, therefore, occurs in parallel with both the rise in cortisol concentrations and the increments in tissue mitochondrial biogenesis and ETS complexes in the fetal sheep (Table 1, Fig. 2A, (25)).

When the prepartum increase in T_3_ is abolished by the surgical removal of the thyroid gland in fetal sheep, mitochondrial content, the abundance of all ETS complexes and ATP-synthase are reduced in the skeletal muscle at term alongside the lower *ANT1* expression compared to the sham-operated controls (Fig. 3; (29)). The total OXPHOS capacity and rates of both pyruvate and fatty acid supported OXPHOS are also reduced in the *biceps femoris* of thyroidectomised (TX) fetuses close to term (Fig. 3B). When data were combined from the TX and sham-operated groups, the muscle mitochondrial content and the OXPHOS rates correlated positively with both the fetal cortisol and T_3_ concentrations during late gestation (29). Partial correlation analysis showed that both hormones were equally important in regulating the mitochondrial content but that the T_3_ was the dominant factor in regulating the muscle OXPHOS capacity during late gestation (29). Similarly, there is a positive relationship between T_3_ concentrations in the cord blood and placental mtDNA content in human infants at birth (92). Thyroidectomy of fetal sheep also reduced the mitochondrial content and *UCP1* expression in perirenal adipose tissue close to term (31). It also lowered the expression of the deiodinase, DIO1, which converts T_4_ to T_3_, in line with the known role of thyroid hormones in regulating the activity of tissue deiodinases in adulthood (93). In contrast, there was THR upregulation and overrepresentation of the biological pathways of fatty acid metabolism in the adipose tissue of the TX sheep fetuses near term (31). In ovine brain, the effects of fetal TX were region specific with decreased CI abundance and pyruvate supported OXPHOS in the cortex and reduced total and CII supported OXPHOS with no alterations in the ETS complex abundance in the cerebellum (32). However, TX had no effect on the mitochondrial content in either the brain region of fetal sheep but led to differential changes in the expression of *ANT1* and the THR and GR receptors in the two regions (32). Fetal TX reduced expression *ANT1* and GR in the cerebellum but not the cortex while, conversely, THR expression was higher in the cortex but not the cerebellum near term (32). In hypothyroid pregnant sows, mitochondrial OXPHOS capacity was reduced in the liver and specific skeletal muscles of their piglets in late gestation (42). Similarly, near term, there were reductions in the mitochondrial content and abundances of complexes II to V solely in the male placenta and in the cardiac complex IV activity in both sexes of pups of hypothyroid rat dams (94, 95). Conversely, administration of thyroid hormones has also been shown to increase the accumulation and oxidative use of fatty acids by the ovine cardiac myocytes (96).

Collectively, the studies manipulating the thyroid and glucocorticoid exposure *in utero* suggest that T_3_ may have a more prominent role in regulating fatty acid oxidation, while glucocorticoids have more direct actions on the availability and metabolic use of carbohydrates during the perinatal period (15). They also indicate that T_3_ is likely to mediate some of the effects of the glucocorticoids on mitochondrial maturation during late gestation in a tissue-specific manner via the glucocorticoid-induced increases in specific tissue deiodinases and THR abundance (13, 15, 89). Indeed, the 4–5-fold increment in plasma T_3_ at birth in response to cold exposure may explain the major neonatal increase in the OXPHOS capacity seen in several tissues at birth (Table 1; Fig. 2C; (29, 35)). However, there are differences in the effects of thyroidectomy and adrenalectomy on muscle mitochondria in fetal sheep with decreases in the ETS complex abundance with thyroid hormone deficiency but not when the prepartum rise in both cortisol and T_3_ are abolished by fetal adrenalectomy (Fig. 3C). This suggests that T_4_ may also have effects on fetal mitochondrial function as T_4_ concentrations are reduced by thyroidectomy but not adrenalectomy of fetal sheep (29, 30). Indeed, during late gestation, the whole body rates of O_2_ consumption and glucose oxidation by fetal sheep are positively correlated with the fetal T_4_ not T_3_ concentrations (44). Indeed, T_4_ and T_3_ are known to have different affinities for the THR isoforms, which, in turn, have distinct affinities for the response elements of the downstream target genes involved in the metabolic responses to environmental conditions (97). In addition, fetal TX slows the prepartum activation of the hypothalamic–pituitary–adrenal (HPA) axis, which leads to lower than normal cortisol concentrations in the TX fetal sheep near to term (29, 98). The effects of T_4_ on OXPHOS may be either direct on the mitochondria or permissive in providing the precursor for T_3_ synthesis or may act via maturation of the fetal HPA or other endocrine axes, including the tissue THR and GR abundances and their isoforms (31, 33, 98). Indeed, the abundance of the receptors for T_3_ and T_4_, their transporters and of the specific deiodinases that metabolise thyroid hormones to their more and less biologically active forms vary between feto–placental tissues and with gestational age and glucocorticoid exposure (13, 97, 99). Indeed, gene deletion of the specific deiodinases that decrease and increase tissue T_3_ availability in brown adipose tissue of fetal mice leads to down- or early upregulation of the adipocyte *UCP1* and *PGC1α* expression, respectively (100, 101). In addition, the adverse effects of thyroid hormone deficiency *in utero* on placental growth and nutrient transfer may also affect the mitochondrial OXPHOS capacity through alterations in the availability of O_2_ and nutrients (15, 95, 102). The interactions between the thyroid and glucocorticoid hormones in regulating mitochondrial maturation are, therefore, complex and may also involve other metabolic hormones with direct actions on ETS function or indirect effects via the cellular availability of OXPHOS substrates.

### Other hormones

Manipulations of cortisol and thyroid hormone concentrations in fetal sheep during late gestation are known to alter the fetal concentrations of insulin, leptin and insulin-like growth factor-1 (IGF-1) and components of their downstream signalling pathways in specific tissues (14, 103). Fetal leptin and insulin concentrations are increased after TX but reduced in adrenalectomised sheep fetuses (15). In contrast, plasma IGF-1 levels in fetal sheep are reduced in hypothyroid conditions and unaffected by fetal cortisol manipulations, although there are tissue specific changes in the IGF-1 expression in response to the altered fetal concentrations of thyroid and glucocorticoid hormones that depend on gestational age (14, 15, 103).

Maternal hormones other than the thyroid and glucocorticoid hormones may also influence the feto–placental mitochondrial development. For instance, continuous maternal infusion of prolactin during rat pregnancy has been shown to increase UCP1 abundance in adipose tissue of the pups by term (104). Moreover, there is growing evidence for sexual dimorphism in the response of feto–placental mitochondria to environmental challenges, which suggests that sex steroids may also be involved in mitochondrial development *in utero* (86, 105). Sex-linked differences in mitochondrial biogenesis, dynamics and abundance of ETS complexes have been observed in the feto–placental tissues of several species in response to fetal growth restriction and chronic hypoxia and with direct maternal glucocorticoid administration (47, 48, 65, 86, 87, 105). In adulthood, oestrogen, androgen and progesterone receptors have all been localised in the mitochondria and can regulate the transcription of mitochondrial genes in both the nuclear and mtDNA (4). Indeed, testosterone overexposure has been shown to reduce the mitochondrial and ATP content of the rat placenta *in vivo* at term and in both rat and human trophoblast cells *in vitro* (106, 107). Conversely, maternal oestrogen is essential during late gestation to maintain the normal development of mitochondrial structure and ATP synthesis in the skeletal muscles of fetal baboons (108). Consequently, the mitochondria in the placenta, fetal adrenal glands and gonads are not only involved in sex steroid synthesis *per se* but may also be regulated functionally by these hormones during intrauterine development.

## Postnatal consequences of the prenatal endocrine environment

Effects of manipulating glucocorticoid and thyroid hormones on mitochondrial OXPHOS before birth are known to persist after birth in several species (Table 3). The postnatal changes include altered mitochondrial content, ETS complex abundance and activity, OXPHOS capacity and the efficiency of ATP production (Table 3). The mitochondrial changes programmed *in utero* are tissue and substrate specific and, particularly in rodents, have been shown to be associated with an increased incidence of metabolic dysfunction later in adulthood, including impaired insulin sensitivity, glucose tolerance, thermogenesis and cellular bioenergetics (2, 5, 73, 108, 114, 115, 116). When cortisol concentrations are raised in fetal sheep within the physiological range before the normal prepartum increment, there are reductions in the abundances of CI to CIV inclusive in the *semitendinosus* muscle, accompanied by increased fatty acid-supported OXPHOS rates in young adulthood (28). However, there were no changes in the ETS complex abundance or OXPHOS capacity in the *biceps femoris* of these adult animals nor in their whole body glucose tolerance or insulin sensitivity (28, 117). In several species, adverse environmental conditions that increase the feto–placental glucocorticoid exposure have also been shown to alter the postnatal mitochondrial function in a number of tissues with potential consequences for age-related mitochondrial diseases (114, 115, 116, 118, 119, 120). In addition, early life environmental programming, particularly of the oocyte, has implications for the inheritance of mitochondrial phenotype and the incidence of inherited mitochondrial OXPHOS dysfunction (120, 121).

**Table 3:** Effects of prenatal glucocorticoid and thyroid manipulations on postnatal mitochondrial OXPHOS capacity.

	Species	Offspring tissue	Offspring age	Postnatal mitochondrial OXPHOS effects	Reference
**Fetal manipulations**					
Cortisol administration	Sheep	Skeletal muscle	10 months	Muscle specific	(28)
125–130 dGA				↓ CI to CIV protein abundance	
				↑ Fatty acid supported OXPHOS	
**Maternal manipulations**					
Synthetic glucocorticoid administration in late pregnancy	Rat	Hippocampus	4 weeks	Abnormal mitochondrial ultrastructure	(109)
				↓ Mitochondrial membrane potential	
				↑ UCP3 protein abundance	
				↓ Mitochondrial ATP content	
	Rat	Frontal cortex	10 weeks	↓ CII, CIV and CV protein abundance	(110)
				↓ CI OXPHOS	
				↓ UCP2 protein	
				↓ Tissue ATP content	
	Rat	Heart	12 weeks	↓ Mitochondrial membrane potential	(111)
				↓ ATP production	
	Rat	Brown adipose tissue	4 months	↓ Mitochondrial content	(60)
				↓ Mitochondrial cristae	
				↓ *PGC1α* expression	
				↓ UCP1 abundance	
				↓ *VDAC* expression	
	Sheep	Adipose tissue	Newborn-6h	↑ UCP1 content	(64)
				↑ VDAC protein	
				↑ Cytochrome c protein	
Maternal cortisol administration in late pregnancy	Sheep	Heart	2 weeks	↑ Mitochondrial ribosomal and CV gene expression	(112)
				↓ *MFN2* gene expression	
Maternal thyroid hormone deficiency in late pregnancy	Rat	Cerebral cortex	15 days	↓ CI activity	(113)
				↓ CI & CII OXPHOS	
	Rat	Heart	56 days	↓ CIV activity	(94)
	Pig	Liver	1 day	↓ Mitochondrial content	(42)
				↓ CIV activity	
		Skeletal muscle		↓ CIV activity	

OXPHOS, oxidative phosphorylation; UCP, uncoupling protein; VDAC, voltage-dependent anion channel, electron transfer system complexes CI to CV; *MFN*, mitofusin gene; *PGC1α*, peroxisome proliferator-activated receptor-γ coactivator gene; dGA, days gestational age.

## Conclusions and future perspectives

**Figure 5:**
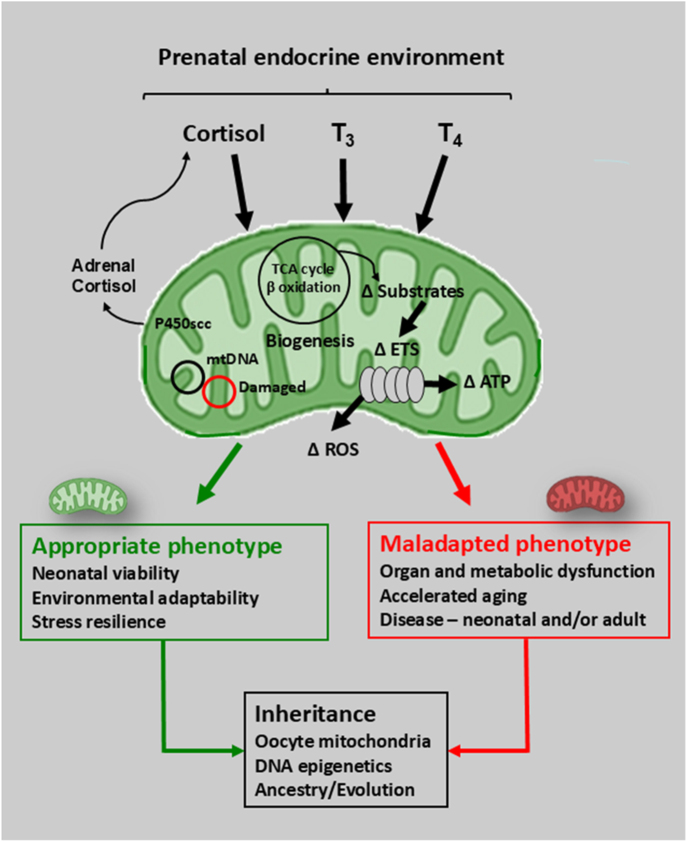
Schematic summary diagram showing the effects of cortisol and thyroid hormones on feto–placental mitochondria during intrauterine development and their subsequent consequences for the mitochondrial phenotype in both individuals and across generations. T_4_ = thyroxine, T_3_ = tri-iodothyronine, ETS = electron transfer system, ROS = reactive oxygen species, mtDNA = mitochondrial DNA, red circle = damaged mtDNA, Δ = change.

Glucocorticoid and thyroid hormones act as key developmental, maturational and environmental signals in regulating the mitochondrial OXPHOS capacity before birth. They are essential for the normal development of mitochondrial structure and function *in utero* and for preparing tissues for the increased energy demands that occur at birth (Fig. 5). By altering OXPHOS and other mitochondrial functions, such as ROS and steroid production, these hormones ensure feto–placental metabolic adaptability, thereby optimising the intrauterine growth and neonatal survival during changes in the substrate and O_2_ availability. However, to date, little is known about the effects of cortisol, T_3_ or T_4_, on mitochondrial dynamics, mitophagy or ETS supercomplex formation before birth. Abnormal exposure to these hormones before birth is also associated with the alterations in the mitochondrial OXPHOS capacity after birth with the potential beneficial or maladaptive consequences for metabolic fitness depending on the prevailing postnatal environment (Fig. 5). However, it remains unclear whether these postnatal outcomes are due to persistence of the mitochondrial changes induced *in utero* and/or reflect prenatal programming of the endocrine axes regulating mitochondrial phenotype after birth (109, 122). The cellular and molecular interactions between the glucocorticoid, thyroid and sex hormones both before and after birth are emerging as important factors in the sex-linked programming of mitochondrial and metabolic dysfunction in the later life (123). However, further studies are needed to define the role of the prenatal endocrine environment on the tissue specificity and sexual dimorphism of adult mitochondrial function with ageing, and to identify the molecular pathways by which this programming occurs. The extent to which an abnormal prenatal endocrine environment leads to acquired mitochondrial diseases in later life or to the inter- and trans-generational inheritance of mitochondrial phenotype also remains largely unknown. Whatever the mechanisms involved prenatal endocrine regulation of mitochondrial phenotype clearly has an important role not only in energetic flexibility but also in the environmental responsiveness and stress resilience throughout life with longer term evolutionary implications for survival of the species (Fig. 5).

## Declaration of interest

The authors declare that there is no conflict of interest that could be perceived as prejudicing the impartiality of the work reported.

## Funding

Writing this review did not receive any specific grant from any funding agency in the public, commercial or not-for-profit sector. Research carried out by the authors at the University of Cambridge cited in this review was funded in part by grants from the Medical Reseach Council, and the Biotechnology and Biological Sciences Research Council.

## Author contribution statement

ALF and AJM conceived and wrote the article. KLD and EJC provided data for the figures. AJF contributed to the ideas and structure of the article, including the figures. All authors approved the final version of the manuscript.
